# Study of the omnidirectional photonic bandgap for dielectric mirrors based on porous silicon: effect of optical and physical thickness

**DOI:** 10.1186/1556-276X-7-391

**Published:** 2012-07-13

**Authors:** Augusto David Ariza-Flores, Luis Manuel Gaggero-Sager, Vivechana Agarwal

**Affiliations:** 1 Department of Physics, Facultad de Ciencias, UAEM, Av., Universidad 1001 Col. Chamilpa, Cuernavaca, 62210, Morelos, Mexico; 2 CIICAp, UAEM, Av., Universidad 1001 Col. Chamilpa, Cuernavaca, 62210, Morelos, Mexico

**Keywords:** Omnidirectional mirrors, Porous silicon, Multilayered mirrors, Dielectric mirrors

## Abstract

We report the theoretical comparison of the omnidirectional photonic bandgap (OPBG) of one-dimensional dielectric photonic structures, using three different refractive index profiles: sinusoidal, Gaussian, and Bragg. For different values of physical thickness (PT) and optical thickness (OT), the tunability of the OPBG of each profile is shown to depend on the maximum/minimum refractive indices. With an increase in the value of the maximum refractive index, the structures with the same PT showed a linear increment of the OPBG, in contrast to the structures with the same OT, showing an optimal combination of refractive indices for each structure to generate the maximum OPBG. An experimental verification was carried out with a multilayered dielectric porous silicon structure for all the three profiles.

## Background

Omnidirectional mirrors (OM) can reflect all the incident light independent of the incidence angle, within a certain wavelength range [1-10]. Omnidirectional properties have been shown using one-dimensional photonic crystals [1], cladded superlattice structures [2], multilayered heterostructures [3], ternary photonic bandgap materials [4], etc. for different systems (for example, Na_3_AlF_6_/Ge, SiO_2_, BaF_2_/PbS, GaAs, etc.) [3-5,7]. Due to their potential applications in optical telecommunications and light-emitting systems, OMs from SiO_2_, polypropylene, Si, GaN, etc. [11-13] have been reported. Several groups have fabricated OMs from porous silicon (PS) in the near-infrared range due to their advantage over metallic mirrors of being non-absorbing and non-dispersive [14-18]. Usually, PS multilayered structures are designed by alternating low- and high-porosity layers like a Bragg mirror [14] or a mechanically stable, gradually varying Gaussian-like periodic profile [15,16]. However, for a required physical thickness and omnidirectional photonic bandgap (OPBG), the best choice of the refractive index profile and the combination of indices are still not known. In this work, we report a comparative study of the dependence of OPBG as a function of maximum refractive index for three different refractive index profiles: sinusoidal, Gaussian, and Bragg type. The comparison was carried out between the structures with the same optical thickness (OT) and physical thickness (PT). An experimental verification was performed with the help of PS multilayered photonic structures.

## Methods

All PS multilayered structures were prepared through anodic etching of a (100)-oriented p-type crystalline Si wafer (resistivity 2 to 5 m*Ω*cm), under galvanostatic conditions [19]. For the electrochemical anodization process at room temperature, the electrolyte mixture was 1:1 (*v*/*v*) of HF (48 wt.%)/ethanol (98 wt.%), respectively. The current density and the etching duration of each layer were controlled by a computer-interfaced electronic circuit where the current density varied from 8.8 to 327 mA/cm^2^, corresponding to the refractive indices of 2.5 and 1.48, respectively. All the structures consisted of 40 periodic unit cells with a sinusoidal, Gaussian, or Bragg refractive index profile. The reflectivity measurements were carried out with a PerkinElmer Lambda 950 UV/VIS spectrophotometer with a variable angle accessory, Universal Reflectance Accessory (URA; Waltham, MA, USA), for 8° and 68°. The maximum and minimum values of the incidence angle were limited due to the angular range covered by URA.

### Theoretical overview

The theoretical simulations of the reflectivity spectra were done using the transfer matrix method for a *p*-polarized electromagnetic wave [20]. Briefly, we suppose that an incident *p*-polarized electromagnetic wave (*E*_*I*_ and *H*_*I*_) passes through a thin multilayered structure. At the first interface (*I*), part of the light reflects and the rest is transmitted. We can relate these light beams using the contour conditions for an incident electromagnetic wave at the interface. The transmitted wave has a phase shift by the time it reaches the next surface (*E*_*II*_ and *H*_*II*_ ); then, in this new surface (*II*), we relate again the reflected and transmitted electromagnetic beams and connect each layer with a transfer matrix:

(1) $$\left[\begin{array}{l} E_{I}\\ H_{I} \end{array}\right]=\left[\begin{array}{ll} \cos(k_{0}h) & i\sin(k_{0}h)/Y_{I}\\ Y_{I}i\sin(k_{0}h) & \cos(k_{0}h) \end{array}\right]\left[\begin{array}{l} E_{\mathrm{II}}\\ H_{\mathrm{II}} \end{array}\right],$$

where *k*_0_ is the magnitude of the wave vector, *h* is the optical path, and *Y*_*I*_ is a function of the refractive index (*n*_*I*_) and the transmitted angle (*θ*_*I*_):

(2) $$Y_{I}=\sqrt{\frac{\varepsilon_{0}}{\mu_{0}}}n_{I}/\cos(\theta_{I}).$$

By making the same procedure, we can couple the electromagnetic field of each interface with the preceding one:

(3) $$\left[\begin{array}{l} E_{I}\\ H_{I} \end{array}\right]=M_{I}\left[\begin{array}{l} E_{\mathrm{II}}\\ H_{\mathrm{II}} \end{array}\right].$$

For the second interface, the electromagnetic field (*E*_*II*_, *H*_*II*_) can be related to the third interface (*E*_*III*_, *H*_*III*_) by

(4) $$\left[\begin{array}{l} E_{\mathrm{II}}\\ H_{\mathrm{II}} \end{array}\right]=M_{\mathrm{II}}\left[\begin{array}{l} E_{\mathrm{III}}\\ H_{\mathrm{III}} \end{array}\right].$$

Then, incident field (*E*_*I*_, *H*_*I*_) can be related to the third field (*E*_*III*_, *H*_*III*_) by multiplying the transfer matrices *M*_*I*_ and *M*_*II*_, resulting in

(5) $$\left[\begin{array}{l} E_{I}\\ H_{I} \end{array}\right]=M_{I}M_{\mathrm{II}}\left[\begin{array}{l} E_{\mathrm{III}}\\ H_{\mathrm{III}} \end{array}\right].$$

In general, if *P* is the number of layers, each one with a specific value of refractive index *n* and optical path *h*, then the first and last interface fields are related by

(6) $$\left[\begin{array}{l} E_{I}\\ H_{I} \end{array}\right]=M_{I}M_{\mathrm{II}}\mathrm{..}M_{P}\left[\begin{array}{l} E_{\left(P+1\right)}\\ H_{\left(P+1\right)} \end{array}\right].$$

The characteristic matrix of the complete system is the result of multiplying each individual 2×2 matrix:

(7) $$M=M_{I}M_{\mathrm{II}}\mathrm{..}M_{P}=\left[\begin{array}{ll} m_{11} & m_{12}\\ m_{21} & m_{22} \end{array}\right].$$

Finally, the total transfer matrix can be reduced to the reflection and transmission coefficients, and the equation can be reformulated in terms of contour conditions. Hence, the reflectivity is given by

(8) $$\;R=r^{2},$$

where

(9) $$\;r=\frac{Y_{0}m_{11}+Y_{0}Y_{s}m_{21}-m_{12}-Y_{s}m_{22}}{Y_{0}m_{11}+Y_{0}Y_{s}m_{21}+m_{12}+Y_{s}m_{22}}$$

and

(10) $$\;Y_{s}=\sqrt{\frac{\varepsilon_{0}}{\mu_{0}}}n_{s}/\cos(\theta_{s}).$$

We used Equation 8 to compute the reflectivity spectrum for a multilayered dielectric structure. The refractive index profiles were obtained from the following equations:

For sinusoidal,

(11) $$\begin{array}{lll} n_{i}= & \frac{n_{\max}-n_{\min}}{2}\sin\left(\frac{2\mathrm{\Pi P}}{N}i-\frac{\Pi }{2}\right)\; & \;\\ & +\frac{n_{\max}+n_{\min}}{2}\;i=\{0,\ldots ,440\}.\; \end{array}$$

For Gaussian (for one period),

(12) $$\;n_{i}=\left\{\begin{array}{ll} n_{\min} & \;i=0\\ \left(n_{\max}\;-\;n_{\min}\right)e^{-d^{2}{\left(i-11\right)}^{2}/\sigma^{2}}\;+\;n_{\min} & \;\;i\;=\;\{1,\ldots ,21\}, \end{array}\right)$$

and for Bragg type,

(13) $$\;n_{i}=\left\{\begin{array}{ll} n_{\max} & \;i=2k\\ n_{\min} & \;i=2k+1, \end{array}\right)$$

where *n*_max_ and *n*_min_ are the maximum and minimum refractive indices, respectively, *P* is the number of periods, *N* is the number of layers, *i* is the label representing an arbitrary layer within a certain interval, *d* is the width of each layer, and *σ*^2^ is the variance.

## Results and discussion

**Figure 1:**
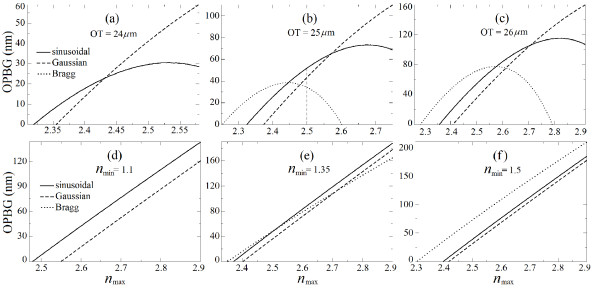
**OPBG as a function of *n*_max_ for the sinusoidal, Gaussian, and Bragg profiles.** Panels (**a**), (**b**), and (**c**) correspond to the OT of 24, 25, and 26 *μ*m, respectively. Panels (**d**), (**e**), and (**f**) correspond to the PT = 7.76 *μ*m for three different values of minimum refractive index. The OPBG was computed between 0° and 85° of incidence angle at 99% of the reflectivity.

Figure 1 shows the comparison of OPBG as a function of maximum refractive index (*n*_max_), for the structures with sinusoidal, Gaussian, and Bragg refractive index profiles for different OT and PT.

The *n*_max_ was varied from 2.2 to 2.9, while the miminum refractive index (*n*_min_) was adjusted to keep the OT constant as (a) 24, (b) 25, and (c) 26 *μ*m. The computed range of *n*_max_ was limited by the experimental capability to obtain high refractive indices (keeping PS as a possible reference material) and the adjusted values of *n*_min_ to keep the same OT of all the structures. Figure 1a,b,c demonstrates that for each OT, one can find a particular value of *n*_max_ at which the profile corresponding to the higher value of OPBG changes. For example, in Figure 1b, the largest OPBG for *n*_max_ range of 2.25 to 2.45, the Bragg-type profile has to be the preferred choice. For 2.45 <*n*_max_ < 2.57, the sinusoidal profile has the largest OPBG, but the Gaussian profile prevails for *n*_max_ > 2.57. A similar behavior is observed for higher OTs (Figure 1c). For the OT of 24 *μ*m, the Bragg-type profile fails to demonstrate any OPBG (Figure 1a). Although the Gaussian structure shows the largest OPBG, the corresponding value of *n*_max_ is also very high.

Figure 1d,e,f shows the comparison of the OPBG for the structures with the same PT, i.e., 7.76 *μ*m. The *n*_max_ was varied from 2.3 to 2.9, while the *n*_min_ was kept constant as (a) 1.1, (b) 1.35, and (c) 1.5. Figure 1a,b,c demonstrates that the Gaussian refractive index profile always requires higher refractive index values to obtain the same OPBG as compared to the sinusoidal refractive index profile. Equivalently, the OPBG obtained for the sinusoidal profile is always higher as compared to that for the Gaussian profile for a given *n*_max_. In spite of the failure of the Bragg-type profile to demonstrate any OPBG for *n*_min_ = 1.1 (see Figure 1d), the tunability to increase/decrease the OPBG for *n*_min_ = 1.35 as compared to the sinusoidal and Gaussian profiles is shown in Figure 1e. One can identify three particular intervals for the Bragg profile (2.35 <*n*_max_ < 2.51, 2.51 <*n*_max_ < 2.72, and 2.72 <*n*_max_ < 2.9) at which the OPBG is higher/lower as compared to the sinusoidal and Gaussian profiles (Figure 1e). For a higher *n*_min_, Figure 1f shows a significant enhancement for the Bragg-type structure, revealing a larger OPBG as compared to the other profiles. Hence, one can obtain the tunability of the OPBG in a certain refractive index range, depending on the available refractive indices and the profile of the photonic structure.

The result shows that no particular profile can be designated as the best profile for the complete range of maximum refractive indices discussed in this work. Apart from that, one can obtain the tunability of the OPBG in a certain refractive index range, depending on the available refractive indices and the profile of the photonic structure. The vertical dashed line in Figure 1b corresponds to *n*_max_ = 2.5 and the particular OT incorporated in the forthcoming experimental and simulated results.

**Figure 2:**
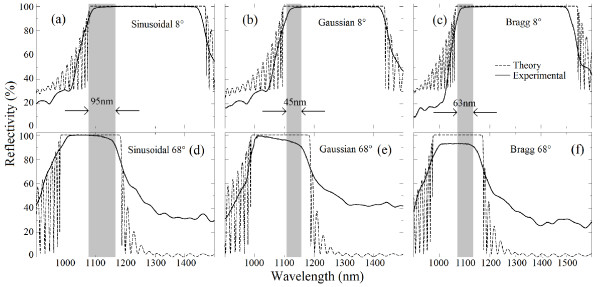
**Theoretical (dashed line) and experimental (solid line) reflectivity spectra for structures with the same OT.** The PBG generated using the (**a**) sinusoidal, (**b**) Gaussian, and (**c**) Bragg profiles for 8°. The corresponding reflectivity spectra at 68° are shown in panels (**d**), (**e**), and (**f**), respectively. The overlapping of the experimental PBG between 8° and 68° is shown as a gray band (taken for the reflectance more than or equal to 90%).

Figure 2 shows the experimental (fabricated with PS multilayers) and simulated reflectivity spectra for the three types of photonic structures at 8° and 68° of incidence angle. As mentioned earlier, the results are obtained for *n*_max_ = 2.5 and 25 *μ*m of OT (dashed vertical line in Figure 1b). OPBG is shown as a vertical gray band. Good agreement between the calculated (dashed line) and the experimental spectra (solid line) is observed. The experimental OPBG was taken with more than 90% of the reflectivity for each multilayered structure. The sinusoidal profile (Figure 2a,d) shows a 95-nm photonic bandgap, while the Gaussian (Figure 2b,e) and Bragg (Figure 2c,f) profiles show 45 and 63 nm of OPBGs, respectively. Hence, for the given value of OT (25 *μ*m) and *n*_max_ (2.5), the sinusoidal profile was shown to have almost twice the OPBG than the other two profiles under discussion.

**Figure 3:**
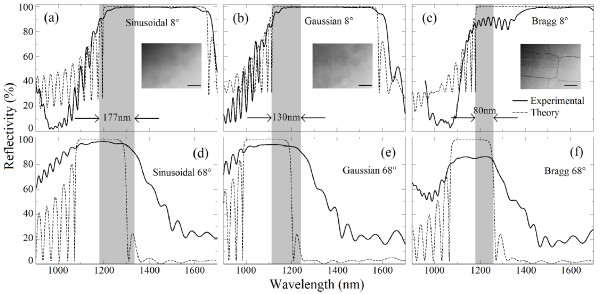
**Theoretical (dashed line) and experimental (solid line) reflectivity spectra.** (**a**) Sinusoidal, (**b**) Gaussian, and (**c**) Bragg refractive index profiles at 8°. The corresponding reflectivity spectra at 68° are shown in panels (**d**), (**e**), and (**f**), respectively. The intersection of the PBG between 8° and 68° is shown as a gray band. The *n*_min_ and *n*_max_ were 1.2 and 2.4, respectively, and the total physical thickness was 7,760 nm for each structure. The inset image shows an optical microscopy surface zone for (a) sinusoidal (b) Gaussian and (c) Bragg profiles. The scale bar corresponds to 200 *μ*m.

On the other hand, Figure 3 shows the experimental and theoretical results for the photonic structures with the same PT. A good agreement is observed between theoretical and experimental results. The overlapping of PBG for different angles was measured as 177 nm for the sinusoidal profile (Figure 3a,d), while the Gaussian (Figure 3b,e) and Bragg (Figure 3c,f) profiles show an OPBG of 130 and 80 nm, respectively. To verify the mechanical stability of such structures, the surface images of the PS multilayered structure corresponding to each profile are shown as insets. The surface fractures observed on the Bragg-type structure (see inset in Figure 3c) are attributed to the high-porosity contrast between two consecutive layers [21-23]. For the sinusoidal and Gaussian refractive index profiles, the inset images (see inset in Figure 3a,b) show a flat-uncracked surface due to the gradual variation of the porosity between consecutive layers, which helps in reducing the stress and enhances the mechanical stability [21]. Therefore, a significant reduction in the intensity of the reflectivity spectra observed for the Bragg-type photonic structure (Figure 3c,f), as compared to the theoretical simulations, is attributed to the cracked structure which provokes a higher dispersion of the incident light.

**Figure 4:**
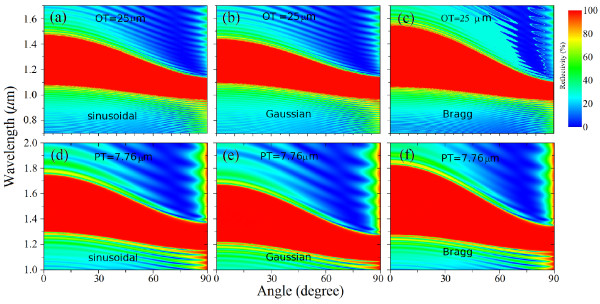
**Contour plot of the reflectivity spectra as a function of the angle and wavelength.** (**a**, **d**) Sinusoidal, (**b**, **e**) Gaussian, and (**c**, **f**) Bragg refractive index profiles. The color scale indicates the reflectivity percentage from 0% (blue) to 100% (red).

Figure 4 shows the theoretical contour plots for the reflectivity spectra as a function of the wavelength and the incident angle for the sinusoidal (Figure 4a,d), Gaussian (Figure 4b,e), and Bragg (Figure 4c,f) mirrors. Figure 4a,b,c corresponds to the photonic structures with the same OT, while Figure 4d,e,f corresponds to the photonic sutructures with the same PT. As the angle of incidence is increased, the PBG (red region) decreases for all the photonic structures. In spite of the largest PBG at 0° (over the other profiles) for the Bragg mirror, the ability for keeping a *semi-constant* stop band, independent of the incident angle, is better demonstrated for the sinusoidal and Gaussian structures, showing a more pronounced fall of the PBG (after 45°) for the Bragg structure, as compared to the other mirrors. Hence, depending on the application, the refractive index profile can be selected to have a larger PBG within a certain angular range (e.g., from 0° to 45°, Bragg mirrors are a better choice) or a small PBG but for any possible incidence angle.

## Conclusions

We demonstrate that the width of the OPBG depends on the choice of the maximum, the minimum, and the difference of the refractive indices for any given profile (sinusoidal, Gaussian, or Bragg-type refractive index profiles). The structures with the same OT showed an optimal combination of refractive indices to generate the largest OPBG, as compared to the structures with the same PT which showed a linear increase in the OPBG. An experimental verification performed with the nanostructured porous silicon dielectric multilayered structures confirmed the superiority of the sinusoidal profile over the Gaussian profile to enhance the OPBG and reduce the structural stress compared to the Bragg structure. This study can be useful to design the required OPBG structures for photonic applications.

## Competing interests

The authors declare that they have no competing interests.

## Authors’ contributions

ADA carried out the theoretical simulations, experimental fabrication, and measurements of the samples. LMGS participated in its coordination. VA conceived the study, worked on the manuscript with AD, and participated in its design and coordination. All authors read and approved the final manuscript.

## Authors’ information

ADA is a Ph.D. student (in Physics) registered at the Faculty of Sciences, UAEM and doing his research work at CIICAp-UAEM, Mexico. LMGS is a professor investigator at the Faculty of Sciences, UAEM and working on the electronic properties of semiconductors from a theoretical point of view. VA is working as a professor investigator at CIICAp UAEM in the field of nanostructured silicon (fabrication, characterization, and applications).
